# *Corrigia vitta* (Dujardin, 1845), the neglected helminth of European rodents

**DOI:** 10.1017/S0031182025101157

**Published:** 2026-01

**Authors:** Jerzy M. Behnke, Neil J. Morley, Joseph A. Jackson

**Affiliations:** 1 School of Life Sciences, University of Nottingham, University Park, Nottingham, UK; 2 Department of Biological Sciences, Royal Holloway, University of London, Surrey, UK; 3 School of Science, Engineering and Environment, University of Salford, Manchester, UK

**Keywords:** *Apodemus sylvaticus*, bank vole, *Clethrionomys glareolus*, *Corrigia vitta*, Digenea, Trematoda, wood mouse

## Abstract

The digenean trematode, *Corrigia vitta*, is a frequently reported component species in studies of helminth communities of wild rodents in Europe, especially those of wood mice and bank voles. It has been known since Dujardin first described the species in 1845, and yet its life cycle is still poorly defined, although Dicrocoeliidae typically have at least 3 hosts in their life cycles. Here, we review the history of nomenclature changes of the species, morphological studies, definitive mammalian host species range and evidence for the identity of intermediate hosts. We also review the epidemiology of *C. vitta*, searching for commonalities between studies that have assessed the effects of intrinsic and extrinsic variables on both prevalence and abundance of the species in wood mice. Furthermore, we identify gaps in knowledge and propose key objectives for future work on the species. We emphasize that if the life cycle of *C. vitta* could be established in the laboratory and maintained in laboratory mice, as a hepatopancreatic specialist in its definitive host, the parasite may turn out to be the source of novel medicines for the treatment of human pancreatic/liver diseases.

## Graphical abstract

**Figure:**
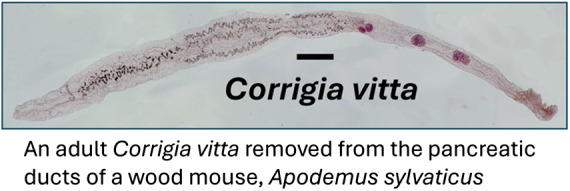

## Introduction

The helminth fauna of small mammals found in the British Isles and on the European mainland is well known (Lewis 1987; Feliu et al. 1997; Sáez-Durán et al. 2021; Lewis et al. 2023 and references cited therein). The life cycles of the dominant rodent-infecting nematode genera *Heligmosomoides, Heligmosomum, Syphacia* and *Aspiculuris* have all been thoroughly described and are well documented in the literature (Durette-Desset 1968; Lewis 1968a, 1987; Bryant 1973; Lewis and D’Silva 1986). The species in these genera are all directly transmitted, either following a brief spell as free-living organisms (*Heligmosomoides* spp., *Heligmosomum* spp.) or without a free-living stage, via rapidly embryonating eggs passed from one infected host to another (*Syphacia* spp.) or via eggs with temporary residence in the environment (*Aspiculuris* spp., *Trichuris* spp.). In contrast, much less is known about the life cycles of helminths that employ intermediate hosts (e.g. Cestoda, Digenea and even some Nematoda). It is perhaps surprising that, given more than a century and a half of research on the helminths of wood mice and bank voles, the full life cycle of one of the species that is often dominant in wild rodent populations is still so poorly known.

The digenean species *Corrigia vitta* belongs to the family Dicrocoeliidae and is often reported to show high prevalence in wood mouse populations (Tables 1 and 3; Langley and Fairley (1982) recorded a prevalence > 90% in June). It inhabits an unusual location in its host and has a life cycle that is still incompletely documented. Nevertheless, the development of more model species, facilitating laboratory exploration as well as field studies, is urgently required in order to deepen our understanding of the Trematoda (Poulin 2025), and this widespread European species has potential in this regard. Here, we trace the history of changes in the nomenclature of this species and highlight studies that have reported components of its life cycle. We also review the epidemiology of *C. vitta*, searching for commonalities between studies that have assessed the effects of intrinsic and extrinsic variables on both prevalence and abundance of the species in wood mice, and we evaluate the influence of extrinsic and intrinsic factors on these measures of infection. Finally, we identify key questions that merit research attention.

## Historical background of nomenclature changes

In his magnum opus, Dujardin (1845) first reported this species as *Distoma vitta*, providing an incomplete description because he only examined the posterior portion of 1 worm extracted from the small intestine of a wood mouse in France. He dealt with the species in section 4 of his monograph, concerned with the *Brachylaimus*-like digenea (p 418 of Dujardin 1845). In his reference list of trematode parasites of British mammals, in a section entitled ‘Unclassified species’, Nicoll (1927) also referred to this species as *D. vitta*.

The genus *Lyperosomum* (Dicrocoeliidae, Odher, 1910) was erected half a century later by Looss (1899). Fragmented sections of the worms, collected by Elton from wood mice trapped near Oxford (reported later in Elton et al. 1931), were examined by Baylis (1927), who moved the species to *Lyperosomum*, and the worm then became *L. vitta*, but not for long. Following Travassos (1944), the species was moved next to the newly created genus *Orthorchis*, as *O. vitta.* This change in genus was not recognized by some subsequent authors for over a decade (e.g. Thomas 1953; Dawes 1968, both referred to *L. vitta*). However, issues of priority were raised by Dollfuss (1954), who drew attention to the work of Strom (1940). The latter author had revised the genus *Lyperosomum* and had erected *Corrigia* for species with the specific characters attributed by Travassos to *Orthorchis*. The parasite is thus currently known as *Corrigia vitta* (Dujardin 1845) Strom 1940.

## Descriptions of *Corrigia vitta*

**Figure 1.**
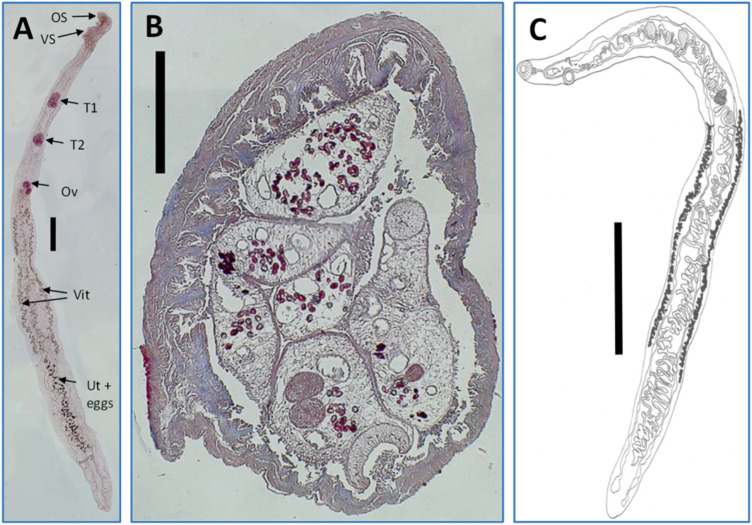
*Corrigia vitta*, adult worms. A. Complete worm extracted from the bile duct of a wood mouse from Surrey (labels are as follows: OS, oral or anterior sucker; VS, ventral sucker; T1, anterior testis; T2, posterior testis; Ov, ovary; Vit, vitelline glands; Ut + eggs, uterus containing eggs). B. Transverse section through a pancreatic duct showing 5 worms lying alongside one another. C. Whole mount drawn by camera lucida under 42 mm. Objective and ×10 ocular lenses. Image copied from Harvey and Channon (1956). A and B are images taken from microscope slides in the collection of the late Prof John W. Lewis, held at the Department of Biological Sciences, Royal Holloway, University of London (with permission of his family). A was stained with borax carmine stain and B with haematoxylin and eosin stain. Scale bars in A and B, 0·5 mm, and in C, 2·0 mm.

Baer (1932) provided the first detailed drawing of the anatomical arrangements of *C. vitta*, and a good drawing of the worm is also provided by Harvey and Channon (1956). We have included this drawing in Figure 1C, as well as a photograph of a stained worm extracted from an infected wood mouse, caught in Surrey. Both show clearly the 2 testes located anteriorly, and the ovary located more posteriorly. The asymmetrically developed vitelline glands lying laterally on both sides of the worms, with one extending further in a posterior direction than the other, are also evident, as is the uterus containing eggs. Harvey and Channon (1956) tabulated a list of dimensions of key morphometric characters of *C. vitta* and compared their values to those provided by some earlier workers (Table 1 in Harvey and Channon 1956).

**Table 1.** Selected studies of the occurrence of *Corrigia vitta* in wood mouse (*Apodemus sylvaticus*), yellow-necked mouse (*A. flavicollis*) and Ural mouse (*A. uralensis*) populations

Author	Location	Loc characteristics	Sample size^a^	Prevalence (%)	Abundance	Intensity	Range
Elton et al. 1931	Oxfordshire	Bagley wood	692	0·86	Ng	Ng	Ng
Harvey and Channon 1956	Exeter	Woods, gardens	34	38·2	1·09	2·85	1–8
Lewis 1968a	Aberystwyth	Rough grassland	133	15·04	1·893	12·59	Ng
Lewis 1968a	Aberystwyth	Woodland	119	35·29	2·891	8·190	Ng
Clark and Fairley 1971	Ireland, 11 counties	Not stated	87	1·14	0·011	1	1
Lewis and Twigg 1972	Surrey	Woodlands	137	19·7	0·94	4·8	1–16
Langley and Fairley 1982	W. Ireland, Galway	Woodland and grassland	323	62·0	^d^	^d^	1–162
O’Sullivan et al. 1984	Ireland, Killarney	Woodland, mixed	170	31·7	Ng	Ng	1–49
Montgomery and Montgomery 1988	N. Ireland	Deciduous woodland	3045	0–77·92	0–10·95	Ng	Ng
Tenora et al. 1991	Denmark	Mixed wood	13^b^	7·7	0·15	2	2
Behnke et al. 1999	Surrey	Woodland	134	17·9	1·1	6·14	1–22
Abu-Madi et al. 2000	SE England, 3 sites	Woodlands, hedges, grassland	399	5·3	0·35	6·65	1–25
de Bellocq Jg et al. 2003	Sicily, Italy	Island	33	33·3	2·0	5·9	1–25
Milazzo et al. 2005	S. Italy, Calabria	Beech, fir forest	697	1·4	0·073	5·1	1–14
Klimpel et al. 2007a	Germany, Westphalia	Not described	27^b^	33·3	4·15	12·4	1–38
Klimpel et al. 2007a	Germany, Westphalia	Not described	33	21·2	1·67	7·9	1–24
Milazzo et al. 2010	S. Italy, NE. Calabria	Med maquis, olive groves	106	1·9	0·066	3·5	3–4
Debenedetti et al. 2015	Spain, Navarre, Erro valley	Forest and pastures	136	16·0	0·5	2·0	1–15
Debenedetti et al. 2016	Spain, Navarre, Erro valley	Forest and pastures	14^b^	21·4	0·29	1·33	1–2
Loxton et al. 2016	Ireland, Galway	Deciduous woodland	152	11·1 & 20·5	Ng	12·4 & 5·56	Ng
Loxton et al. 2017	Ireland, Galway, Wicklow and Dublin	Deciduous woodland	389	47·0	1·0–14·0	Ng	Ng
Stuart et al. 2020	Ireland	Various	512	33·8	4·4	13·0	Ng
Kirillova et al. 2024	Russia, Mordovia	Mixed forests	822^c^	0·4 & 1·3	0·12 & 0·02	Ng	1–46
Marsh et al. 2024	Oxfordshire	Wytham wood, mixed deciduous	50	14	2·0	14·3	1–52

a Apodemus sylvaticus

b Yellow-necked mice, *Apodemus flavicollis.*

c Ural field mouse, *Apodemus uralensis.*

d Monthly values, from June to May, for the number of parasites per mouse are provided in the paper.

Ng = not given in the paper.

**Figure 2.**
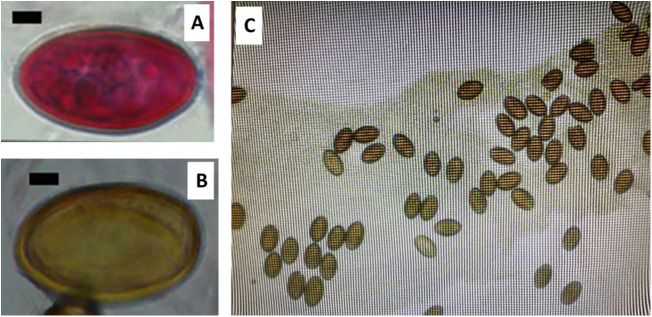
Eggs of *Corrigia vitta*. A. Egg stained by haematoxylin and eosin stain. B. Unstained egg. C. Compressed posterior section of an adult worm, with released eggs. A and B are images taken from microscope slides in the collection of the late Prof John W. Lewis, held at the Department of Biological Sciences, Royal Holloway, University of London (with permission of his family). Scale bars in A and B are 5 μm.

In particular, eggs are small, typically brown in colour, with a smooth surface. They are operculated at 1 pole and when discharged from the adult parasite are embryonated, i.e. contain a fully developed miracidium (Figure 2; Baer 1932; Ashour 1995). Recorded egg size can vary between different sampling locations (Baylis 1927; Baer 1932; Harvey and Channon 1956), although it has been suggested that a ‘normal’ size may be approximately 37–38 μm length and 18–23 μm width (Harvey and Channon 1956). Such potential variation in size makes diagnosis using egg morphology unreliable, and consequently, infections are typically determined through dissection.

More recently, the morphology and cytochemistry of the adult parasite have been studied at the ultrastructural level (Robinson and Halton 1982, 1983, 1984; Magee et al. 1993; Ashour 1995). In particular, tegument structure has been found to be highly specialized. The distribution and forms of mitochondria in the tegument suggest that the ventral surface of the parasite, which is in close approximation with the host pancreatic duct brush border, may be functionally specialized for absorption at the relatively high local oxygen tension that may occur in this microanatomical context during host feeding cycles. On the other hand, the dorsal tegument may be adapted for energy metabolism during prevailing periods of anaerobiosis (Robinson and Halton 1983, 1984).

## Taxonomic identity and related species

Based on Dawes (1968), *Corrigia vitta* is currently assigned to the family Dicrocoeliidae Odher, 1910 (subfamily Dicrocoeliinae, Looss 1899). The Dicrocoeliidae comprise over 400 described species, which are hepatopancreatic specialists living predominantly in the liver, gall bladder, pancreas and associated ducts. Most parasitize birds and mammals, including domestic animals (Otranto and Traversa 2002; Manga-González et al. 2024), but some species also have reptiles and marsupials as definitive hosts. One characteristic of this family of trematodes is that their life cycles involve 2 intermediate hosts and can be completed in terrestrial environments without access to water (Pojmańska 2008). The family contains many genera, including species that parasitize rodents in Africa (e.g. *Paraconcinnum leirsi*, Ribas et al. 2012) and S. America (e.g. *Platynosomoides lunaschiae*, Martins et al. 2022).

Other *Corrigia* spp. have also been described, and mostly from birds, including Phasianidae (*C. petrowi* Sultanov 1961, in the rock partridge, *Alectoris graeca; C. corrigia* in black grouse, *Lyrurus tetrix*; Tizzani et al. 2021), Anatidae (*C. obscura*, Daniels and Freeman 1976 in the N. American black duck, *Anas rubripes*) and Numididae (*C. vulturini*, Lori and Balbo 1990 in vulturine guineafowl, *Acryllium vulturinum*).

**Figure 3.**
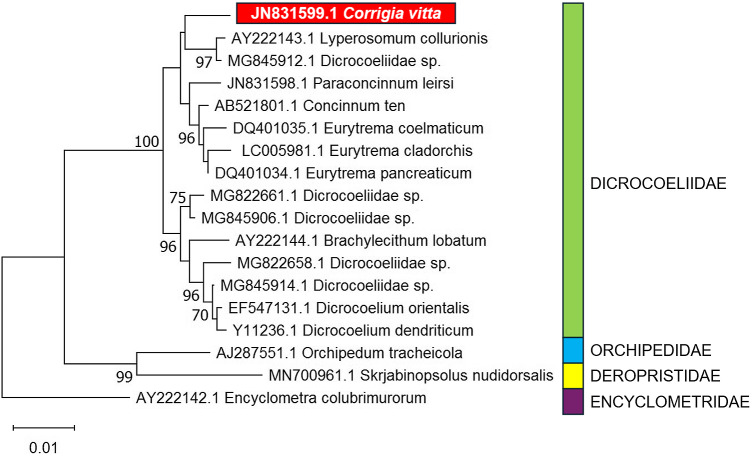
Updated phylogenetic analysis of dicrocoeliid trematodes based on 18S rDNA, showing the position of *Corrigia vitta*, employing longer sequences (>1400 bp) currently available in GenBank (see Ribas et al. 2012, for previous analysis). Where more than 1 long sequence was available for a nominal species, a single arbitrarily selected sequence was included per species. Sequences for members of other plagiorchiid families are included as outgroups. Analysis was carried out via the Maximum Likelihood method (following Ribas et al. 2012) in MEGA11 (Tamura et al. 2021), employing a general time reversible model (Nei and Kumar 2000) with invariant sites. Initial trees were obtained via the BioNJ method applied to pairwise distances estimated by Maximum Composite Likelihood (MCL). The inferred tree (log likelihood −3286) is shown with evolutionary distances indicated via the scale (substitutions per site). Bootstrap support (Felsenstein 1985) for clusters is indicated where this is greater than 70% (based on 1000 replicates). The analysis was based on 1434 base positions, excluding all positions with gaps or missing data.

The only molecular sequence data of reliable provenance that we are aware of for *Corrigia* is a single 18S rDNA sequence obtained from *C. vitta* (Ribas et al. 2012; GenBank ref, JN831599, 1770bp). Based on 18S rDNA sequences, Ribas et al. (2012) identified 2 genetic clades in the Dicrocoeliidae, with *C. vitta* clustering with *Concinnum, Eurytrema, Lyperosomum* and *Parasconcinnum* spp. The second clade incorporated *Dicrocoelium* and *Brachylecithum* spp. However, a similar analysis (Figure 3), to which more recent 18S rDNA sequences for other dicrocoeliid species have been added, returns *C. vitta* as a singular lineage within the Dicrocoeliidae not falling within any significant subfamilial cluster. This may be a function of the limited amount of information in the currently available 18S rDNA sequences to estimate increasingly complex trees, and is indicative that multi-locus analyses may be required in future to resolve the genus-level relationships of *Corrigia* within the family.

## Life cycle

The full life cycle of *C. vitta* has not been determined, although much speculation exists in the literature. Some authors have promoted the *Dicrocoelium dendriticum* life cycle as a suitable template for *C. vitta*, going so far as to suggest that ants may also act as second intermediate hosts for this species (Canning et al. 1973; McCarthy et al. 2023). However, there is no evidence to support this analogy.

The *C. vitta* life cycle has not been satisfactorily resolved experimentally, with only the studies of Schmidt (1967, 1969) providing tentative, if not conclusive, evidence of the hosts involved. The Dicrocoeliidae family have 3 or 4 hosts in their life cycles. It has proved possible within this family to differentiate amongst known life cycles a number of types related to taxa, subfamilies or genera (Niewiadomska and Pojmańska 2011). The genus *Corrigia* is considered to be related to *Eurytrema* (see Figure 3), with both having daughter sporocysts that are highly specialized and secondarily altered (Galaktionov and Dobrovolskij 2003). Experimental studies of a number of species within these 2 genera have allowed the determination of a ‘Type-*Eurytrema*’ which encompasses a molluscan first intermediate host, an arthropod second intermediate host and a terrestrial mammal definitive host. This type possesses xiphidiocercariae, with only a rudimentary tail, that do not emerge from the sporocyst (Pojmańska 2008; Niewiadomska and Pojmańska 2011).

Within the genus *Corrigia*, these life cycle patterns have been most extensively studied for *Corrigia corrigia* (Panin and Romanenko 1978; Žďárská 1986). The adults of this species reside in the pancreas of gallinaceous birds. Eggs are released into the environment within faeces and consumed by a terrestrial snail intermediate host. Cercariae develop in daughter sporocysts, which lack a birth pore. Once the cercariae are fully developed, the sporocyst migrates through the snail’s tissues until it reaches the mantle cavity, where it is expelled into the environment. A well-developed endocyst surrounds the sporocyst, protecting it from climatic conditions. An isopod second intermediate host ingests the cyst (*Hemilepistus fetschenkoi* and *Orthometopon planum* in Panin and Romanenko 1978), and once within the body of the host, the cercariae penetrate the tissues and develop into metacercariae. Transmission to the definitive host occurs through predation on isopods as part of their normal diet (Panin and Romanenko 1978). A detailed account of the structural and histochemical characteristics of the transmission stages from the snails to the isopod second intermediate host is given by Žďárská (1986).

Schmidt (1967, 1969) studied the trematode parasites of terrestrial snails over a number of years in a habitat in which wood mice (*A. sylvaticus*) were commonly infected with *C. vitta.* The snail *Clausila bidentata* was frequently infected with Dicrocoeliidae sporocysts containing xiphidiocercariae with rudimentary tails. A second mollusc, *Cochlodina laminata*, was also infected less frequently by a morphologically similar parasite. Both of these snail species have also been recorded as hosts of *C. vitta* in the Ukraine (Iskova et al. 1995; Gibson et al. 2005). Although Schmidt (1967, 1969) failed to find naturally infected isopods among 523 that he examined, when he experimentally fed infected *C. bidentata* and *C. laminatas* to woodlice (*Philoscia muscorum, Porcellio scaber*), Dicrocoeliidae metacercariae were recovered from 30% of fed isopods. Two wild *A. sylvaticus* caught from a different habitat, where no natural *C. vitta* infections had been recorded, were fed on metacercarial cysts and specimens of *C. vitta* were subsequently found in the pancreas of these mice. However, Schmidt (1967, 1969) was unable to completely eliminate the possibility of a pre-existing infection in these wild-caught mice, raising questions about the reliability of his experiments, and so far, no follow-up experiments have been undertaken. Nevertheless, the studies of Schmidt (1967, 1969) follow the life history pattern of the ‘Type-*Eurytrema*’, as would be expected, and consequently woodlice remain a strong possibility as the most likely and commonest second intermediate host of *C. vitta*. However, it may be that in different geographical regions, *C. vitta* exploits other arthropods as second intermediate hosts, particularly in habitats where woodlice may be absent or rare. In N. Ireland, where the prevalence of *C. vitta* varied between deciduous and coniferous woodland sites, isopods were never recorded in the diet of wood mice from these habitats, based on their stomach contents (Montgomery 1982; Montgomery and Montgomery 1990a). However, increased consumption of arthropods in their food is consistent with *C. vitta* being more common among the mice from coniferous woodland, which fed more on animal food, compared with those from a deciduous site where seeds dominated the diet (Montgomery and Montgomery 1988, 1990a)

## Mammalian definitive hosts of *C. vitta*

In Europe, a common host of *C. vitta* is the wood mouse, *A. sylvaticus*, but it has also been recorded from the yellow-necked mouse (*A. flavicollis*; Debenedetti et al. (2016), Ural mouse (*A. uralensis*; Kirillova et al. 2024; Makarikov et al. 2017) and Black Sea field mouse (*A. ponticus*), Makarikov *et al*., 2017; (Table 1).

Bank voles (*Clethrionomys glareolus*) are susceptible to infection with *C. vitta*, but generally, prevalence and abundance are low in this species (Table 2), and in some sites, *C. vitta* has been recorded as absent from bank voles, while prevalent in sympatric wood mouse populations (Clark and Fairley, 1971; Tables 1 vs 4). Lewis (1968b) recovered low-intensity infections from bank voles living in rough grassland sites and in the woodlands near Aberystwyth, Wales, but not on Skomer Island (Table 1). O’Sullivan et al. (1984) recorded infections in bank voles in Ireland but concluded that *C. vitta* occurred only rarely in bank voles, which concurs with Canning et al. (1973), working in Devon, SW England, who recorded only 1 infected bank vole in their sample of 57 animals. Milazzo et al. (2003a) recovered *C. vitta* from just 3 bank voles from their sample of 193 wild rodents from S Italy (Table 2), but prevalence and abundance in wood mice were also very low in Calabria (Tables 1 and 2), indicating that this region was not a good transmission zone for *C. vitta*. There are also records of *C. vitta* from *Microtus agrestis* in England (Lewis and Twigg 1972; Canning et al. 1973), but other studies of the helminth fauna of *Microtus* spp. have not recorded *C. vitta* as a parasite of voles of this genus (e.g. see Jackson et al. 2014 for *M. agrestis* in the UK).

**Table 2.** Selected studies of the occurrence of *Corrigia vitta* in bank and grey-sided vole populations

Author	Location	Loc characteristics	Sample size	Prevalence	Abundance	Intensity	Range
Lewis 1968b	Aberystwyth	Rough grassland	29	10·3	0·310	3	1–9
Lewis 1968b	Aberystwyth	Woodland	65	10·8	0·442	4·1	
Lewis and Twigg 1972	Surrey	Woodlands	88	5·7	0·125	2·2	1–5
Canning et al. 1973	Slapton Ley, Devon	Woodlands, reed beds	57	1·75	0·175	10	Ng
O’Sullivan et al. 1984	Ireland, Killarney	Woodland, mixed	140	1·4	Ng	Ng	1–4
Haukisalmi et al. 1987	Finland, north	Taiga, peatlands, alpine habitats	532^a^	0·5	Ng	Ng	Ng
Tenora et al. 1991	Denmark	Mixed wood	46	2·2	0·087	4	4
Milazzo et al. 2003a	S. Italy, Calabria	Beech, fir forest	193	1·55	0·026	1·66	1–2
Ribas et al. 2009	N.E. Spain, Catalonia	Montane forests	249	3·32	0·100	3·11	1–7
Lynggaard et al. 2018	Denmark, Kongelunden	Not described	21	19·0	1·52	8·0	3–14

a *Clethrionomys rufocanus*, grey-sided vole.

Ng = not given in the paper.

*Mus* species may also harbour *C. vitta,* but reports are very few. In the Navarra region of N Spain, Sainz-Elipe et al. (2004) recorded *C. vitta* in *Mus spretus*, the Mediterranean mouse, although both prevalence and abundance were very low (1% and 0·11, respectively). We found 1 report of *C. vitta* from house mice and black rats on Corsica (Milazzo et al. 2003b, citing unpublished theses [Esteban, 1983 and Piqueras 1992] from the University of Valencia). However, *C. vitta* has not been reported from *R. rattus, R. norvegicus* and *M. musculus* in other studies of these commensal rodents from neighbouring regions of Europe and further afield (Tattersall et al. 1994; Alfonso-Roque 1995; Casanova et al. 1996; Kataranovski et al. 2008; Feliu et al. 2012; Stojcevic et al. 2004; Juhász et al. 2024; see also Table 4).

Relatively high prevalence rates of *C. vitta* have been recorded in garden dormice (*Eliomys quercinus*) on the islands of Majorca, Menorca and Formentera, in the Balearic Archipelago in the Mediterranean (Table 3; Esteban et al. 1987). In a subsequent survey of 502 small mammals on Formentera, the garden dormouse was the only host species infected with *C. vitta* (Mas-Coma et al. 1998). Algerian hedgehogs (*Atelerix algirus*), wood mice, house mice and black rats were also examined, but none of these carried *C. vitta* (Tables 3 and 4), perhaps suggesting that this may have been a different species of *Corrigia*, or a dormouse–infective genetic variant.

**Table 3.** Records of *C. vitta* in wood mice, bank voles and other hosts or in which few additional details were given

Author	Host	Sample size	Location	Information provided
Baylis 1928	*A. sylvaticus*	Not stated	Near Oxford	Just a species list
Thomas 1953	*A. sylvaticus*	15	Ulva, Hebrides	One specimen in 2 pieces from upper small intestine. Morphometric details given
Sharpe 1964	*A. sylvaticus*	280	Woods near Bristol	Recorded only as *Corrigia* sp.
Corbet and Southern 1977	*A. sylvaticus*	Not stated	British Isles	Focus on the rodents in British Isles
	*C. glareolus*			
Cordero Del Campillo et al. 1980	*A. sylvaticus*	Not stated	Spain, Andalusia	Species list
	*C. glareolus*			
	*Mus musculus*			
	*Mus spretus*			
	*E. quercinus*			
Montgomery 1980	*A. sylvaticus*	495	N. Ireland	Species list with locations in hosts
Genov 1981	*A. flavicollis*	Not stated	Bulgaria	Species lists and descriptions
Feliu et al. 1987	*A. sylvaticus*	691	Spain, 52 sites in Pyrenean region	Species list
Esteban et al. 1987	*E. quercinus*	Majorca 110, Menorca 55, Formentera 110	Spain	Prevalence = 45·5% in Majorca, 49·1% in Menorca, 33·6% Formentera
Montgomery and Montgomery 1989	*A. sylvaticus*	Not stated	N. Ireland	Helminth community analyses
Johnston et al. 1990	*A. sylvaticus*	Not stated	N. Ireland	Pathology in the pancreas
Magee et al. 1993	*A. sylvaticus*	Not stated	N. Ireland	Worms used for cytochemical observations
Iskova et al. 1995	*A. flavicollis*	Not stated	Ukraine	List of parasites from rodent hosts
Feliu et al. 1997	*A. sylvaticus*	Not stated	Spain	List of parasites from rodent hosts
	*M. spretus*			
	*C. glareolus*			
	*E. quercinus*			
Mas-Coma et al. 1998	*Eliomys quercinus*	164	Spain, Formentera	Lists of parasites from rodent hosts
Arabul et al. 2024	*A. sylvaticus*	Not stated	Georgia	Checklist

**Table 4.** Selected studies of helminths in rodent populations, with no record of *C. vitta*

Author	Host	*n*	Location	Observations
Sołtys 1949	4 species: 140Cg, 30Mar, 16Ms, 24Af.	210	E. Poland, Białowieża	
Furmaga 1957	8 species: 191Aa, 70As, 20Af, 286 Mar, 3 Mag,4Mr, 1Ms, 112Mm	687	Poland, Lublin	
Boyd 1959	*Apodemus sylvaticus hirtensis*	9	Scotland, St. Kilda	Accidental kills, as protected sp. here
Vaucher and Hunkeler 1967	6 species: 61Af, 33As, 25Cg, 3Mn, 1Mar, 10Mag	133	Switzerland	
Bernard 1969	9 species: 589As, 429Cg, 248Rn, 167Mm, 2479Mar, 825Mag, 244Ms, 135At, 72Eq	5188	Belgium	
Kisielewska 1970	*Clethrionomys glareolus*	1467	E. Poland, Białowieża	
Clark & Fairley 1971	*Clethrionomys glareolus*	42	SW Ireland	
Canning et al. 1973	*Apodemus sylvaticus*	58	England, Slapton Ley, Woodlands, reed beds	Only present in 1 bank vole (Table 2)
Wiger et al. 1976	4 species: 460Cg, 6Mag, 12As, 10Crut	488	N and S. Norway	
Murúa 1978	*Apodemus sylvaticus*	81	England, Slimbridge and Bristol	
Murúa 1978	*Clethrionomys glareolus*	38	England, Slimbridge and Bristol	
Mészáros and Murai 1979	9 species: 3Rn, 9Mm, 34Af, 23As, 1Mar, 8Mag, 13Mn,42Cg, 7Ms	140	Rumania	
Tenora et al. 1979	*Clethrionomys glareolus*	398	S. Norway	
Tenora et al. 1983	7 species: 185Cg, 25Crut, 90Cruf, 59Mag, 33Moec, 4At, 3Af	399	Finland, Lapland	
Haukisalmi and Henttonen 1993	*Clethrionomys glareolus*	1244	Finland, north and south	
Tenora and Staněk 1994	5 species: 106Mar, 103Cg, 174Af, 164As, 58Mm	605	Czech Republic, Moravia	
Ryan and Holland 1996	*Apodemus sylvaticus*	40	Ireland, Wicklow	
Galán-Puchades et al. 1998	*Apodemus sylvaticus*	432	S. France and N. Spain	Compares data before and after fires
Mas-Coma et al. 1998	4 species: 164Eq, 147As, 69Mm, 78Rr	458	Spain, Formentera	
Behnke et al. 2001	*Clethrionomys glareolus*	139	NE Poland, 3 sites	
Mažeika et al. 2003	4 species: 120Cg, 12Mag, 7Af, 10Aa	149	Lithuania	
Tenora 2004	*Apodemus sylvaticus*	Ns	Czech Republic	
Bajer et al. 2005	*Clethrionomys glareolus*	250	NE Poland	
Eira et al. 2006	*Apodemus sylvaticus*	557	Portugal, Coimbra, coastal sand dunes	
Klimpel et al. 2007b	*Clethrionomys glareolus*	29	Germany, Westphalia	
Jackson et al. 2009	*Apodemus sylvaticus*	100	C. England, Nottinghamshire	
Ondriková et al. 2010	2 species: 51Af, 96Aa	147	SE. Slovakia	
Bjelic-Cabrilo et al. 2011	*Clethrionomys glareolus*	588	Serbia, Fruska Gora	
Salvador et al. 2011	*Clethrionomys glareolus*	313	France, Ardennes	
Skyrienė et al. 2011	2 species: 103Af, 287Cg.	390	Lithuania	
Bjelić-Čabrilo et al. 2013	4 species: 12Aa, 18As, 2Af, 6Mm	38	NW. Serbia	
Čabrilo et al. 2014	*Apodemus flavicollis*	86	N. Serbia.Peripannonic region	
Grzybek et al. 2015a	*Clethrionomys glareolus*	922	NE Poland, 3 sites	
Vlasov et al. 2015	8 species: 168Au, 17Af, 84Aa, 6Mmin, 3Mm, 123Cg, 83Mar, 6Cm	490	W. Russia	
Loxton et al. 2016	*Clethrionomys glareolus*	177	Ireland, Galway	
Rynkiewicz et al. 2019	*Apodemus sylvaticus*	1674	England, 3 woods near Liverpool	Only by faecal egg counts
Stuart et al. 2020	*Clethrionomys glareolus*	362	Ireland, 6 sites	
Merabet et al. 2021	*Apodemus sylvaticus*	50	N. Algeria, Kabylia	
Behnke et al. 2021	*Apodemus sylvaticus*	483	N. England, N Yorkshire	
Kirillova et al. 2024	*Apodemus flavicollis*	635	Russia, Mordovia	

Ns not stated.

Abbreviations used.

Aa (*Apodemus agrarius*), Af (*Apodemus flavicollis*), As (*Apodemus syvaticus*), At (*Arvicola terrestris*), Au (*Apodemus uralensis*), Cg (*Clethrionomys glareolus*), Cm (*Cricetulus migratorius*), Cruf (*Clethrionomys rufocanus*), Crut (*Clethrionomys rutilus*), Eq (*Eliomys quercinus*), Mag (*Microtus agrestis*), Mar (*Microtus arvalis*), Mm (*Mus musculus*), Mmin (*Micromys minutus*), Mn (*Microtus nivalis*), Moec (M. oeconomus), Mr (*Microtus raticeps*), Ms (*Microtus subterraneus*), Rn (*Rattus norvegicus*), Rr (*Rattus rattus*).

Note that *Microtus raticeps* is considered to be a synonym of *M. oeconomus*, and both are currently known as *Alexandromys oeconomus*.

*Microtus subterraneus* = *Pitymus subterraneus.*

There are also reports of *C. vitta* from water voles (*Arvicola amphibious*) (Gibson et al. 2005; McCarthy et al. 2023) in the British Isles and Spain, as well as from muskrats (*Ondatra zibethica*) from the USSR (Serkowa 1948; Gibson et al. 2005), although more recent studies in Lithuania and Germany failed to find the species in muskrats (Mažeika et al. 2009; Schuster et al. 2021).

## The preferred site of *C. vitta* in hosts and pathology

As in other Dicrocoeliidae, *C. vitta* is a parasite that is adapted for life in the hepatopancreatic system of its mammalian host. Adult worms live in the interlobary ducts of the pancreas of wood mice and bank voles, as described for the first time by Baer (1932; see also Harvey and Channon, 1956). Usually, they can be seen easily through the duct walls when the pancreas is spread out during dissection of the alimentary system, as dark lines running along the length of the ducts, reflecting the dark colour of the eggs in the uteri of the worms (Behnke, *pers. obs*.). Occasionally, worms may be detected in the duodenum or small intestine, perhaps individuals that had been displaced by intense competition in the narrow interlobary ducts or by host resistance, or through senility.

Harvey and Channon (1956) and Schmidt (1969) observed no obvious pathology of the interlobary ducts, other than distention and some thickening of the duct walls when more than 1 worm lay alongside each other (see also Figure 1B). However, both may have been dealing with low-intensity infections (maximum burden was 8 worms). Johnston et al. (1990), on the other hand, observed severe consequences of infection with *C. vitta*, including gross dilation of pancreatic interlobary ducts, epithelial cell hyperplasia, fibrous tissue, lymphatic infiltration and signs of inflammation. They also observed disorganization of the exocrine acini (clusters of cells, forming the functional units that secrete enzymes) in the pancreas, loss of zymogen granules and atrophy, and concluded that *C. vitta* induced partial chronic pancreatic ductal occlusion in wood mice.

There is also strong evidence of an association between *C. vitta* infection and the richness of intestinal microbiota. Marsh et al. (2024) found that microbial species diversity in the ileum and caecum of wood mice was positively associated with the abundance of *C. vitta*. It was speculated that in heavy infections of *C. vitta*, the flow of anti-microbial pancreatic fluids into the gastrointestinal tract may be disrupted, allowing a greater diversity of microbiota to develop (Marsh et al. 2024).

## Epidemiology of *C. vitta*

### Definition of terms

Following Margolis et al. (1982) and Bush et al. (1997), we refer below to the mean intensity of worm burdens as the mean worm burden of mice that were infected with *C. vitta*, and to abundance as the mean worm burden of all the animals in the subset referred to, including uninfected animals. Where authors did not provide data on mean abundance (e.g. Lewis 1968a) but gave the intensity and the number of infected animals, we calculated abundance as ([mean intensity × no of infected mice]/all mice in the data subset). If mean intensity was not provided, we calculated mean intensity as ([mean abundance × total rodents sampled]/no of infected mice in the data subset). Prevalence is given as the percentage of animals harbouring at least 1 worm or shedding at least 1 egg/gram of faeces.

### Methodology of worm removal

It is very difficult to extract whole worms from the interlobary ducts, as they are very fragile and easily tear when grasped with forceps in an attempt to extract them from a duct. Great care and delicate, patient work are required. The difficulties of extracting whole worms were experienced by the early workers on this species (see Baer 1932, who initially located the remainders in the interlobary ducts of the pancreas). Prior to Baer (1932), the pancreas was probably mostly neglected. Elton et al.’s (1931) study reported an extremely low prevalence of worms found only in the upper part of the small intestine, probably because the pancreas was not examined. Our own data reveal that *C. vitta* can have a high prevalence in wood mice from woods from the same region of Oxfordshire (JMB personal observations; and see Marsh et al. 2024).

### Extrinsic factors

#### Geographical distribution of *C. vitta*

*Corrigia vitta* appears to have a patchy distribution across the British Isles (Montgomery and Montgomery 1990b) with most records concentrated in Southern England (Baylis 1928; Elton et al. 1931; Harvey and Channon 1956; Sharpe 1964 [but see by Murúa 1978]; Lewis and Twigg 1972; Behnke et al. 1999; Abu-Madi et al. 2000). In more northerly areas of England our own local surveys in Nottinghamshire have never revealed *C. vitta* here in wood mice or bank voles (Jackson et al. 2009) and studies in Yorkshire and Merseyside have also failed to report the species (Table 4).

However, *C. vitta* has been well documented in Wales, in Ceredigion (Lewis 1968a, 1968b) and in N. Ireland (Montgomery and Montgomery 1988) as well as in the Republic of Ireland (Langley and Fairley 1982; O’Sullivan et al. 1984; Loxton et al. 2017; Stuart et al. 2020). There is only 1 record of *C. vitta* from Scotland, and that was on Ulva in the Inner Hebrides (Thomas 1953).

On the European mainland, there are records from Denmark (Tenora et al. 1991), Germany (Klimpel et al. 2007a), Finland (Haukisalmi et al. 1987), Spain (Debenedetti et al. 2015, 2016), Italy (Milazzo et al. 2003a, 2005, 2010), Georgia (Arabul et al. 2024) and Russia (Kirillova et al. 2024). Equally fascinating are studies that have failed to find *C. vitta*, some of which were located in regions where others have found the parasite (Compare records in Tables 1–3 with those in Table 4).

It has been suggested that this patchy distribution is associated with the geographical distribution of molluscan intermediate hosts (Schmidt 1969; Mas-Coma and Montoliu 1978). Certainly, the distribution in the UK of *C. bidentata*, the potential snail host proposed by Schmidt (1967, 1969), is not uniform, preferring woodland habitats and calcareous environments. Although common in the southeast of England, its occurrence becomes more restricted towards the north of the country, particularly in Scotland (Kerney 1999).

#### Site-dependent variation in infections

Some authors have studied infections in rodents sampled from different sites within a local region. O’Sullivan et al. (1984) utilized 20 trap sites, clustered in 5 ecologically distinct habitat types and found that there was a significant difference in the prevalence of *C. vitta* between these in wood mice. In woodlands with light and heavy ground cover prevalence was 55% and 31%, respectively, and in sites with dense grass, bracken and brambles 26%, while no infected mice were recovered from sites with rhododendron dominating, and without ground cover.

Perhaps the largest study in this field was by Montgomery and Montgomery (1990b), who reported on helminth burdens from wood mice trapped at 2 sites where long-term monitoring was implemented and also at 15 additional sites across N. Ireland. *Corrigia vitta* showed a patchy occurrence, with high prevalence and abundance in some sites, but was totally absent from others. Abu-Madi et al. (2000) found that 28·1% of wood mice from the Isle of Wight carried *C. vitta*, but only 2·3% at the Egham site and none were infected at Dungeness. Abundance was also significantly higher in mice from the Isle of Wight (2·1 worms/host) compared to the other 2 sites (0·2 and 0 at Egham and Dungeness, respectively.

In an interesting variant on this theme, Loxton et al. (2017) studied infections in wood mice in regions of Ireland that were free of bank voles and those that had been invaded by bank voles and found that the abundance of *C. vitta* was significantly higher in the absence of bank voles. While the prevalence was overall 20% in 3 sites, the 1 exception was Coole Nature Reserve in Galway, which is located in a bank vole invaded region where no worms all of this species were recorded. However, in the other bank vole invaded site, Merlin Park, *C. vitta* was prevalent and with an abundance similar to those in the 2 bank vole non-invaded sites. Stuart et al. (2020) found a highly significant site effect for *C. vitta*, for wood mice sampled from 9 sites across Ireland, representing the original introduction core sites for bank voles (3 sites), the invasion front (3 sites) and sites that were still free of bank voles (3 sites). Interestingly, the parasite was totally absent from wood mice sampled at locations in Limerick, in the core sites close to Foynes Port, which is believed to be where bank voles were introduced into Ireland from Germany in the 1920s (Stuart et al. 2007). Abundance in wood mice was significantly higher at the expansion front than at the uninvaded sites.

Several explanations can be offered to explain site effects. For the life cycle of *C. vitta* to be completed and to persist, it is likely that at least 4 taxonomically unrelated organisms must be available locally, and their distributions overlap (the parasite, a molluscan first intermediate host, an arthropod second intermediate host and a mammalian definitive host). In the case of a total absence of a parasite species from the definitive hosts in a site, therefore, the most obvious explanation is that at least one of these taxa is missing locally. The parasite may not have been introduced into the site when colonized by rodents, and/or susceptible intermediate hosts of C. *vitta* have not colonized the site. It may be that in some sites the intermediate molluscan and arthropod intermediate hosts are present, but in such low densities (because the environment is not entirely suitable for their optimal development and survival) that transmission cannot be maintained consistently. In addition to the total absence of *C. vitta* from the Dungeness site (Abu-Madi et al. 2000), infections with *Heligmosomoides polygyrus* also showed very low prevalence and abundance (Abu-Madi et al. 1998). It was suggested that acidification of the soil surface at Dungeness (Ferry and Waters 1988) may have been detrimental to the survival of *H. polygyrus*. High soil acidity may also have been detrimental to the survival of the intermediate hosts of *C. vitta*, although Abu-Madi et al. (1998, 2000) did not monitor the diversity or densities of molluscan or arthropod species in the site (but see Morris and Parsons 1991). Another possibility in the case of wood mice in Ireland is that the absence of *C. vitta* from the core site reflects a dilution effect arising from the introduction and subsequent local proliferation of bank voles (Stuart et al. 2020), although neither Stuart et al. (2020) nor Loxton et al. (2016) found the parasite in bank voles in Ireland. However, like wood mice, the bank voles would have fed on wood lice, but the introduced genotypes may have been more resistant to infection with *C. vitta* than those in other locations where bank voles were found to harbour mature worms (Table 2).

#### Annual variation in infections

Few studies have monitored *C. vitta* infections in rodent populations for more than a year at a time. The earliest study lasting for more than a single year was by Elton et al. (1931), who trapped rodents monthly for almost 4 years in Oxfordshire (from September 1925 until April 1928). However, the prevalence of *C. vitta* in this population was too low to allow a meaningful temporal analysis. Although Montgomery and Montgomery (1988) sampled wood mice monthly from the same sites for almost 3 years (33 months), their analysis of data focused on seasonal changes (see below). Behnke et al. (1999) reported on sampling for 4 years, but only in September of each year, and found that the prevalence of *C. vitta* differed markedly between years. In their study, 1996 was a year with the lowest prevalence and 1997 with the highest, but there was no long-term trend, and it was suggested that the between-year variation in prevalence probably reflected differences in the availability of the arthropod intermediate hosts, possibly influenced by climatic differences between years and availability of predators. As for prevalence, mean abundance also varied significantly between years, from a low in 1994 to a peak in 1997. Climatic conditions can also influence the availability of parasitic infections within molluscan hosts (Morley and Lewis 2008), which may impact annual occurrence in mammalian hosts.

#### Seasonal variation in infections

In W. Ireland in Galway prevalence of infection with *C. vitta* increased steadily from a low in August (≈30%) to a peak in March (≈80%), followed by a dip in April–May, and an even higher peak in June with almost 95% of mice carrying the parasite (Langley and Fairley 1982). Mean worm burdens were steady throughout most of the year, averaging about 8–10/mouse, but peaked in June at ≈ 25 worms/mouse. The fall in prevalence after June was attributed to an influx of uninfected juveniles following the breeding period. Prevalence rose subsequently as gradually more mice became infected, but the lack of a rise in mean worm burdens was attributed to a lack of heavily infected animals at this time of the year compared to June, when 35% of mice had more than 30 adult worms each.

Marked seasonal cycles, indicating cyclic occurrence of *C. vitta,* were recorded by Montgomery and Montgomery (1988) among wood mice trapped in Tollymore and Clandeboye in N. Ireland. Peak worm burdens were recorded in the winter period in Tollymore (February–March) in each of the 3 years, but only in February 1980 at Clandeboye. Although no statistical models were provided for *C. vitta* from bank voles in Catalonia, NE Spain, tabulated data show higher values for prevalence in summer months (7·9%, compared with 3·6% in autumn, 3·5% in winter and 1·7% in spring [Ribas et al. 2009]). The mean intensity of infection was highest in winter (7·5 worms/infected host).

Three studies, involving monthly or seasonal sampling of wood mouse populations, failed to detect any major or significant seasonal variation in infections with *C. vitta*. In County Kerry, in Ireland, *C. vitta* was present in wood mice throughout the year, with prevalence varying from about 10% in April-May to over 40% in September (O’Sullivan et al. 1984), but not showing any marked season-dependent peak. Abu-Madi et al. (2000) also failed to find a significant difference in the prevalence or abundance between seasons, but both prevalence and abundance in these surveys were very low, with a total absence of *C. vitta* from one of the sites (Dungeness). More recently, Stuart et al. (2020) sampled at 9 sites located across Ireland and also did not report seasonal differences of infection parameters as significant, nor any statistical interaction between season and the other factors included in their statistical models.

**Figure 4.**
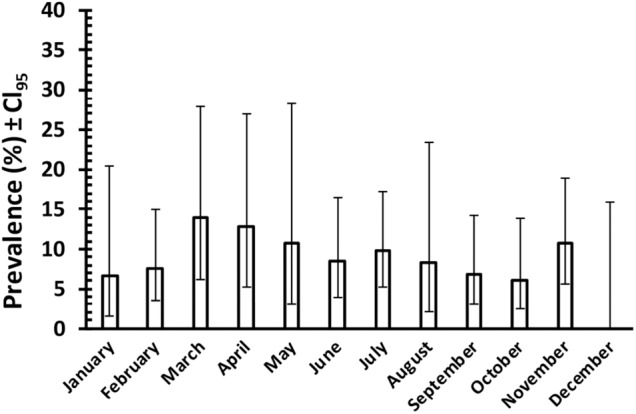
Prevalence of *C. vitta* in adult wood mice in Surrey in the period from August 1975 until February 1978 (inclusive, and both sexes combined). Prevalence is based on faecal *C. vitta* egg counts of mice trapped in the months shown, and released after inspection. The mice were not marked individually; therefore, the data probably include repeated assessments of certain individuals. The number of animals sampled in each month from January to December was as follows: 45, 53, 36, 39, 28, 59, 51, 48, 59, 66; 56, 21. (Unpublished data of J. W. Lewis).

Figure 4 shows the prevalence of *C. vitta*, by month, based on faecal egg counts recorded from 561 samplings of adult wood mice in Surrey in the period from August 1975 until February 1978 (J. W. Lewis unpublished data). Although these data cannot be analysed statistically because the mice were not individually tagged and therefore probably include repeated measures, the difference in prevalence between months varied only from 0 % in December to 13·9% in March, and given the wide 95% confidence limits, this suggests that seasonal variation in prevalence as assessed by monthly values was minimal in this population.

Where dips in prevalence and abundance were found in late summer months (Langley and Fairley 1982; Montgomery and Montgomery 1988), this is most likely attributable to an influx of juvenile, uninfected mice into the population. The longevity of *C. vitta* in its definitive hosts is not known, but the marked age-dependent increase in infection parameters (see below) suggests that the species is long-lived. In Langley and Fairley’s study, transmission probably occurred throughout the year, but this cannot have been the case in Montgomery and Montgomery’s study, in which worm burdens declined to zero in between the peaks they recorded. Despite the wood mouse population size fluctuating with a drop in late spring months, the number of sampled animals throughout this period was still sufficient to allow a realistic estimate of prevalence and abundance. This may indicate that the longevity of *C. vitta* is limited and that after acquisition in late summer, parasite burdens begin to decline in spring, in situations where continued exposure to infection is interrupted. Either way, it is clearly of relevance and reflects the longevity of *C. vitta* (see below).

Unusual, rare weather conditions can also affect the prevalence of *C. vitta* in relevant years. A prolonged period of hot, dry weather in the British Isles in the summer months of 1976 resulted in drought, particularly in the south of England, and was followed by an atypically wet period. Morley and Lewis (2014) found that while wood mice in S. England were free of infections with *C. vitta* in the summer period during the drought, there was a surge of infections in both juvenile and adult wood mice in the autumn following the post-drought heavy rainfall. This was attributed to increased availability of infected molluscan and arthropod intermediate hosts in the recovering ecosystem, benefitting from the prolonged post-drought heavy rainfall.

### Intrinsic factors

#### Age-dependent variation in infections

Helminths are generally regarded as long-lived parasites in their hosts because of their strategies for evading host responses (Behnke 1987; Maizels et al. 2004, 2012, 2018). Not surprisingly, therefore, helminth species richness tends to increase with host age (Montgomery and Montgomery 1989) as does the abundance of long-lived species (Behnke et al. 1999), because hosts accumulate worms that they cannot eliminate throughout their lives.

Although the exact longevity of *C. vitta* is not known, if it were a long-lived species, we would expect both prevalence and abundance to increase with host age. Surveys of wood mice in which there was some focus on age have confirmed this prediction. The first study to quantify differences in the prevalence of *C. vitta* in juvenile and adult wood mice was that of Lewis (1968a) in Wales. Prevalence of infection in adult wood mice was 34·6% in the rough grassland site and 53·1% in woodlands, whilst in juvenile mice the values were 2·5% and 29%, respectively. Langley and Fairley (1982), working in Ireland, also noted a significant difference in prevalence between juvenile (28%) mice and adults (79%), when the borderline between age classes was based on mouse weight (<17·0 g and >16·5 g). Montgomery (1982) found that both intensity and prevalence were positively associated with mean eye lens weight (a correlate of age) among wood mice from Tollymore, where both infection parameters were high. Behnke et al. (1999) found that both prevalence and abundance differed between age classes. Mice in the youngest age class were not infected, but those in the intermediate and oldest age classes showed similar prevalence of *C. vitta* of about 20%. Despite the overall low prevalence of *C. vitta* in the study by Abu-Madi et al. (2000), 18 of the 20 mice with *C. vitta* were adult mice. An age effect was also recorded in wood mice in S. Italy by Milazzo et al. (2005), with prevalence in adult wood mice being 5 times that in juveniles (2·1 vs 0·4%, respectively), although no statistical support was provided. Loxton et al. (2017) found a highly significant difference in abundance between 3 age classes of wood mice in Ireland, with abundance lowest in the youngest age cohort and highest in the oldest, and this relationship was evident among wood mice trapped in bank vole-invaded sites and those without bank voles, in both years of the study.

The data in Table 5 also show a marked and significant difference in prevalence between juvenile and adult wood mice, and this was consistent in both sexes (Unpublished data of J. W. Lewis). However, these data were based on faecal egg counts, and the low values, particularly in juvenile mice, may be partially attributable to the presence of immature worms, which would not have been detected by faecal egg counts.

**Table 5.** Prevalence of *Corrigia vitta* in wood mice from Surrey in the period from August 1975 until February 1978, inclusive, based on faecal egg counts (Unpublished data of J. W. Lewis)

	*n*	No. infected	Prevalence	95% confidence limits
Males^a^	435	29	6·7	4·08–10·62
Females^a^	311	23	7·4	5·02–10·74
Adult males	318	26	8·2	5·66–11·72
Adult females	243	22	9·1	6·66–12·22
Juvenile males	117	3	2·6	0·97–6·20
Juvenile females	68	1	1·5	0·16–7·53
Adults^b^	561	48	8·6	6·87–10·56
Juveniles^b^	185	4	2·2	0·49–7·13
ALL combined	746	52	7·0	5·25–9·14

a Not significantly different: *χ*^2^_1_ = 0·13, *P* = 0·7.

b Significantly different: *χ*^2^_1_ = 7·87, *P* = 0·005.

Taken together, these studies concur and show that an age effect on the prevalence and abundance of *C. vitta* in wood mice is a highly predictable phenomenon. With increasing host age, worms accumulate in wood mice, with the consequence that the values of both parameters are reliably higher in older mice compared to those derived from juveniles. Surveys across the geographic range in which *C. vitta* infections have been observed show that this is a universal aspect of the epidemiology of this parasite in wood mice.

#### Host sex-dependent variation in infections

Many different studies have shown that in mammals, prevalence and abundance of infection with helminths may differ between the sexes (Alexander and Stimson 1988; Zuk and McKean 1996). In most cases, males are reported to be more likely to be infected and to harbour more and larger parasites than females (Poulin 1996a, 1996b), and some studies have attributed male-bias in parasite burdens to host body mass which in sexually dimorphic mammalian species, is often greater in males compared to females (Moore and Wilson 2002; Harrison et al. 2010; Zduniak et al. 2023). Others have attributed male bias to host behaviour, hormonal differences between the sexes influencing immunocompetence and a range of other factors (Schalk and Forbes 1997; Ferrari et al. 2004; Skorping and Jensen 2004; Duneau and Ebert 2012). However, there are also studies showing that in certain situations the bias may be in favour of females harbouring more intense infections (Sanchez et al. 2011; Grzybek et al. 2015b; Bourgoin et al. 2021).

Lewis (1968a) wrote that there was very little difference in the prevalence of *C. vitta* between the sexes. In his rough grassland site, 16% of males and 12·3% of females harboured *C. vitta*, and in the woodland site, 43·4% of males and 20·9% of females were infected. Intensity of infection for male and female mice was 14·1 and 9·0/infected mouse, respectively, in the rough grassland site and 8·1 and 9·6/infected mouse, respectively, in the woodlands. Although Lewis (1968a) did not employ statistical analysis to test these data, numerically, 3 of the 4 values indicate a bias in favour of male mice, and overall, he concluded that male mice were more heavily infected with *C. vitta*. A male bias was also recorded by Loxton et al. (2016, 17·2% prevalence in males and 8·5% in females), but without statistical support. However, intensity was in the opposite direction (7·81 worms/infected male and 14·8 for females).

Three studies failed to find a sex bias in infections with *C. vitta*. No difference between sexes in *C. vitta* infections was found by O’Sullivan et al. (1984, 33% prevalence in males and 30% in females), Behnke et al. (1999, 19·8% prevalence in male and 14·0% in female mice, and for abundance 1·0 and 1·3, respectively), nor by Abu-Madi et al. (2000; 5·7% prevalence in male and 4·8% in female mice, and for abundance 0·37 and 0·33, respectively) in data that were thoroughly analysed by statistical models. The data in Table 5 also show little difference in prevalence between the sexes.

Other studies have found a female bias in prevalence and/or abundance. A sex bias in the prevalence of *C. vitta* in favour of female mice (67% in females and 58% in males) was noted by Langley and Fairley (1982) in Ireland, but was not significant, although the authors explained this bias as female mice requiring more food in the breeding season because of the metabolic demands in pregnancy and lactation. Loxton et al. (2017), who also fitted appropriate statistical models to their data, reported a significant female sex bias, with the abundance of *C. vitta* higher in female mice in both years, although the difference between sexes was greater in 2012 compared with 2011, when the overall abundance of *C. vitta* was higher. Milazzo et al. (2003a) found *C. vitta* only in female bank voles (prevalence = 3·7%. The males were not infected.

Studies of helminth infections in wood mice have generally found that sex-bias is context dependent (Lewis et al. 2023), and taken together, those studies considered above in which prevalence and abundance of *C. vitta* were quantified for each sex, and analysed statistically with other factors taken into account, support this view. Where a difference between the sexes has been detected, it was relatively small and swayed one way or the other depending on how prevalent the parasite was in the population in particular seasons, years and/or sites. This conclusion is consistent with studies in other mammalian hosts subject to parasitism by helminths and arthropod ectoparasites, which have also found significant sex-bias in parasitism to be highly context dependent (Krasnov et al. 2005, 2012; Junker et al. 2022).

## Conclusions and recommendations

Given that *Corrigia vitta* has been known about for so long, it is surprising that the details of its life cycle have still not been fully clarified. Although there are some hints as to the likely molluscan first intermediate hosts, there is still no reliable evidence for their identity. Evidence from work on *D. dendriticum* shows that through dedicated and painstakingly thorough scrutiny of all available molluscs in a vicinity where transmission takes place, it is possible to establish the likely identity of the molluscan host (Manga-González et al. 2001). However, even for that species, experimental evidence is required to complete the work. A similar approach could be adopted for *C. vitta*.

The second intermediate host is also not known with any degree of certainty. As for the molluscan host, therefore, species of arthropods, particularly isopods, living in endemic regions need to be surveyed for the presence of metacercariae (see Panin and Romanenko 1978 for *C. corrigia*). Although there have been indications that woodlice may carry metacercariae, other invertebrates living in transmission zones also need to be carefully scrutinized. Such studies, if based on traditional techniques such as dissection or tissue digestion, require resilience and patience by skilled workers, since undoubtedly the proportion of infected hosts is likely to be extremely low, given the range of possible invertebrate hosts and their population densities. Moreover, all will undoubtedly also harbour other species of helminths. Nevertheless, from a practical point of view, it will often be quicker and cheaper to examine large numbers of arthropod intermediate hosts for Dicrocoeliid metacercariae using the traditional techniques of squashing between 2 glass slides and examining under a dissecting microscope than applying molecular methods. Metacercarial cysts are fairly easy to spot this way, and it is possible to process a lot of material in a few hours. Molecular techniques might then be applied to confirm the identity of metacercariae in the positive samples. Additionally, and perhaps more ambitiously, modern high-throughput molecular methods and automated sample processing could be exploited. For example, homogenization of the whole (relatively small) candidate intermediate hosts could be followed by nucleic acid extraction and PCR, with 1 or more of these steps automated to allow very large sample sizes to be handled efficiently. A preliminary to this would be more genomic information for *C. vitta* (such as a whole genome or mitogenome) to allow greater scope for the design of efficient and specific primers.

Experimental completion of the life cycle could be achieved by first selecting naturally infected wood mice with high egg loads in their faeces. The eggs of *C. vitta* are easily recognizable, and measures of eggs/gm of faeces can be obtained by the McMaster method (M.A.F.F. 1977), if necessary, by replacing saturated salt solution with higher density solutions such as sucrose or 35–40% zinc sulphate. Eggs could then be concentrated by collecting from the surface of the solution and syphoning off the top ml or so into another test tube. By adding water, the density would be reduced, and the eggs can then be concentrated by centrifugation. Locally collected snails could then be fed on appropriate food contaminated with the eggs, and then, a few weeks later, sporocysts should be evident on the dissection of infected snails. Snail species that produce slime balls or extrude sporocysts containing cercariae would then need to be exposed to a range of locally abundant invertebrates, including ants and wood lice, to identify those species that are susceptible to infection by *C. vitta*. Metacercarial cysts should be evident on dissection a few weeks after exposure.

The longevity of *C. vitta* in its definitive hosts is still unknown. Although the age-dependent accumulation of adult worms suggests it is a long-lived species, the seasonal collapse in prevalence and abundance reported by Montgomery and Montgomery (1988) perhaps indicates that it may be a matter of a few months rather than a year or more. Longevity could be determined by housing naturally infected wood mice under animal house conditions and monitoring faecal egg output. Alternatively, uninfected, lab-raised wood mice or captured wild wood mice treated with a suitable anthelmintic could be infected with metacercariae, but this would depend on the life cycle having been established in the laboratory or infected second intermediate hosts being available in the field for the harvest of metacercariae.

Since Sainz-Elipe et al. (2004) recovered worms from *M. spretus*, the Mediterranean mouse, it is possible that other *Mus* species, including laboratory-maintained *M. musculus*, may be susceptible to infection with *C. vitta*. Although we are unaware of any reliable evidence that house mice may be susceptible to *C. vitta* (Milazzo et al. 2003b; Feliu et al. 2012), there is a report of *Corrigia* sp. occurring in both house mice and black rats (*R. rattus*) on Corsica (Milazzo et al. 2003b). However, *C. vitta* was not found in *R. rattus* and *M. musculus* from the nearby islands of Eivissa (Mas-Coma et al. 2000) and Sicily (Milazzo et al. 2003b), nor in other studies of these commensal rodents (Feliu et al. 2012). If house mice and rats (*R. norvegicus*) are found to be susceptible to infection with *C. vitta*, then experimental work on the host/parasite relationship of the adult worms will be possible. Parasites that live in the hepatopancreatic system of their host must be equipped with survival strategies that allow them to persist despite the locally secreted enzymes and products of the immune system, including high concentrations of IgA. Since epidemiological data indicate that worms are likely to be long-lived to some extent, this suggests that the worms employ countermeasures for avoiding the consequences of such host secretions and the effectors of host resistance (immunomodulatory factors; Shepherd et al. 2015; Okakpu and Dillman 2022; Yeshi et al. 2022). These may offer possibilities for developing novel medicines for the treatment of human diseases associated with the liver and pancreas, e.g. autoimmune diabetes (Hernández-Bello et al. 2010; Wang et al. 2017).

In summary, *C. vitta* is undoubtedly a fascinating parasite, but one for which many gaps in knowledge remain to be filled. Here we have reviewed studies that have contributed to the current understanding of its biology, identified questions that warrant research attention and indicated that potentially it may have much to offer for human and veterinary medicine as a study model. We hope thereby to have encouraged others to rise to the research challenges and grasp the research opportunities presented by this enigmatic organism.

## Data Availability

The authors confirm that the data supporting the findings of this study are available within the article. We have also included unpublished data extracted from the laboratory books of Prof John W. Lewis, who passed away in 2022. Following convention, we have obtained the approval of John’s family for the inclusion of these data in this paper.
